# A case of false mother included with 46 autosomal STR markers

**DOI:** 10.1186/s13323-015-0026-y

**Published:** 2015-06-30

**Authors:** Li Li, Yuan Lin, Yan Liu, Ruxin Zhu, Zhenmin Zhao, Tingzhi Que

**Affiliations:** Shanghai Key Laboratory of Forensic Medicine, Institute of Forensic Science, Ministry of Justice, 1347 West Guangfu Road, Shanghai, 200063 China

**Keywords:** STR, SNP, X-STR, mtDNA, Forensic genetics

## Abstract

**Background:**

For solving a maternity case, 19 autosomal short tandem repeats (STRs) were amplified using the AmpFℓSTR^®^ Sinofiler^TM^ kit and PowerPlex^®^ 16 System. Additional 27 autosomal STR loci were analyzed using two domestic kits AGCU 21+1 and STRtyper-10G. The combined maternity index (CMI) was calculated to be 3.3 × 10^13^, but the putative mother denied that she had given birth to the child. In order to reach an accurate conclusion, further testing of 20 X-chromosomal short tandem repeats (X-STRs), 40 single nucleotide polymorphism (SNP) loci, and mitochondrial DNA (mtDNA) was carried out.

**Findings:**

The putative mother and the boy shared at least one allele at all 46 tested autosomal STR loci. But, according to the profile data of 20 X-STR and 40 SNP markers, different genotypes at 13 X-STR loci and five SNP loci excluded maternity. Mitochondrial profiles also clearly excluded the mother as a parent of the son because they have multiple differences. It was finally found that the putative mother is the sister of the biological father.

**Conclusions:**

Different kinds of genetic markers needfully supplement the use of autosomal STR loci in case where the putative parent is suspected to be related to the true parent.

## Findings

### Background

Profiles derived from polymorphic short tandem repeats (STRs) are used worldwide in paternity testing and individual identification. In complex cases of kinship analysis, autosomal single nucleotide polymorphism (SNP), X-chromosomal short tandem repeat (X-STRs), and mitochondrial DNA (mtDNA) could be used to complement autosomal STR typing.

Genotypes of STRs and X-STRs are routinely determined using commercial PCR-based amplification kits with subsequent fragment length determination using capillary electrophoresis with laser-induced fluorescence of labeled primers.

Several years ago, SNP and mtDNA analysis using a PCR and electrospray ionization mass spectrometry-based methods [1, 2] have been developed and reported. For a given individual, the PCR/electrospray ionization time-of-flight mass spectrometry (ESI-TOF-MS)-based assay provides a simple profile consisting of a read-out of 40 binary autosomal SNP markers identified by the Kidd laboratory in 2007 [3]. Meanwhile, this assay offers an efficient high throughput method for profiling the control region of mtDNA that identifies differences between individuals without targeting specific nucleotide positions. This approach provides resolution exceeding that obtained by sequencing the minimum HV1 and HV2 coordinates (16024–16365 and 73–340) by determining the base compositions of 24 short (80–120 bp) amplicons derived from tiling primers covering coordinates 15924–16428 and 31–576 [4, 5].

In this paper we describe an interesting case. A couple went to the Public Security Bureau to declare account for a boy, they claimed the child was abandoned two years ago shortly after birth, and they picked him up and brought him up to date. In order to prove that the boy was really not their child, parentage testing was performed as officially requested. The putative mother (M) shared at least an allele with the boy (B) at each of the 46 autosomal STRs detected, but the putative father was excluded as the biological father with 18 inconsistent loci out of the 46 markers. The mother was adamant that she had not given birth to the child. Below, we emphatically describe the use of additional non-STR genetic markers (SNPs, X-STRs, and mtDNA) to interrogate the potential maternal association between the putative mother and child.

### Methods

Blood samples from the putative mother and the child were collected with informed consent under protocols approved by the IFS ethics committee at the Institute of Forensic Science, Ministry of Justice, China. DNA extraction was performed using a Chelex-100 and proteinase K protocol [6]. The quantity of DNA derived was determined spectrophotometrically and was subsequently aliquoted into the various kits following manufacturer’s guidelines.

Capillary electrophoresis-based STR and X-STR typing was performed on an Applied Biosystems 3130XL genetic analyzer following PCR on an Applied Biosystems GeneAmp 9700 thermalcycler. Data was analyzed using Applied Biosystem’s GeneMapper software V3.2. Commercial autosomal STR panels employed in this work include Applied Biosystem’s 16-marker AmpFℓSTR Sinofiler and Promega’s 16-marker PowerPlex 16. Twenty seven additional autosomal STRs were interrogated using the domestic 21+1 kit [7] and the Typer 10 panel [8]. The commercial X-chromosomal STR panel employed in this work was Mentype® Argus X-8 Kit (Biotype® AG, Germany) [9]. Additional X-chromosomal STRs were interrogated using the in-house IDtyper X-16 kit [10].

Mass spectrometry-based SNP and mtDNA typing was performed using Ibis Biosciences’ PLEX-ID platform [11]. A set of primer pairs to amplify 40 autosomal SNP loci were arranged into a panel of eight 5-plex reactions. Twenty-four primer pairs in eight 3-plex reactions were employed to tile across an extended HV1/HV2 domain of the mitochondrial genome corresponding to coordinates 15924–16428 and 31–576. Genotypes of SNP markers and base compositions (i.e., the number of A’s, G’s, C’s, and T’s) of each amplicon of mtDNA were determined using fully automated high throughput mass spectrometry on PLEX-ID platforms.

### STR analysis

As shown in Table 1, the putative father was clearly excluded as being the biological father because there was 18 inconsistent loci out of the 46 tested autosomal STRs. However, at each of the 46 loci, there was at least one shared allele between the putative mother and the child (Table 1). Based on the allele frequencies of Chinese Han population, the combined maternity index (CMI) of 3.3 × 10^13^ by no means excludes the putative mother from being the biological mother.

**Table 1:** Typing results of 46 autosomal STR loci

STR locus	Mother	Boy	Father	Loci	Mother	Boy	Father
D8S1179	15	10, 15	10, 13	D1S1677	14, 15	10, 15	14, 15
D21S11	30, 31	29, 31	31, 32.2	D11S4463	13, 14	14, 15	14, 15
D7S820	11	11	11, 12	D1S1627	13	13	13, 14
CSF1PO	11, 12	12	10, 11	D3S4529	15, 16	15	13, 16
D3S1358	16, 17	16	14, 15	D2S441	11.3, 12	11.3, 14	10, 12
TH01	9	9	9	D6S1017	10, 12	8, 10	8, 10
D13S317	10, 11	11, 12	8, 11	D4S2408	8, 10	8, 9	9
D16S539	9, 11	11, 12	13	D17S1301	12, 13	9, 13	12, 13
D2S1338	19, 23	19, 23	20, 23	D1GATA113	7, 11	7, 11	7, 11
D19S433	14, 15.2	13.2, 15.2	13.2, 14	D18S853	12, 14	11, 14	11, 13
vWA	17, 19	17, 18	17, 18	D20S482	12, 13	13	14, 15
TPOX	8	8, 11	8, 11	D14S1434	14	14	14, 15
D18S51	13, 16	16, 19	13, 15	D9S1122	12, 13	12, 13	12, 13
D5S818	11, 13	11, 13	11, 12	D2S1776	12, 13	11, 13	9
FGA	23, 24	24, 25	23	D10S1435	13, 14	13, 14	13, 14
Penta D	9, 11	11	12, 13	D5S2500	17	17, 18	14, 18
Penta E	14, 17	12, 17	11, 14	D18S1364	16, 20	16, 20	15
D12S391	18, 22	18, 22	19, 21	D13S325	20, 21	19, 20	19, 21
D6S1043	12	12, 14	18, 19	D2S1772	21, 28	24, 28	24, 30
D6S474	14, 18	17, 18	14	D11S2368	20, 22	18, 20	17, 20
D12ATA63	12	12, 17	17	D22-GATA198	14, 17	17, 18	17, 21
D22S1045	15, 17	15, 16	17	D8S1132	19	19, 22	19, 20
D10S1248	13	12, 13	14, 17	D7S3048	20, 23	23, 27	25, 26

The putative mother refused the conclusion that she was the real mother. In order to get at the facts, the likelihoods of the genotype profiles given various identity-by-descent (IBD) distributions were then calculated by J Ge and B Budowle using MPKin [12, 13]. According to the analysis results, paternal aunt nephew relationship was very likely.

### X-STR analysis

Table 2 depicts the X-STR profiles of the putative mother and the boy at the 20 X-chromosomal STR loci interrogated by capillary electrophoresis. The putative mother was clearly excluded as being the biological mother as her X-STR profile was not consistent with that of the child at 13 out of the 20 X-STR loci detected.

**Table 2:** Typing results of 20 X-STR loci

X-STR locus	Mother (M)	Boy (B)
GATA165B12	10, 12	9
DXS101	24, 26	24
GATA172D05	8, 11	10
HPRTB	12, 14	13
DXS981	13.3, 15	14
DXS8378	11	11
DXS6795	16, 17	16
GATA31E08	9, 11	12
DXS6809	33, 35	34
DXS6803	11, 12	11
DXS9902	10, 11	9
DXS6807	11, 14	14
DXS7423	15	14
DXS7133	9	10
DXS6810	18, 19	18
DXS7132	15, 16	13
DXS10134	37, 38	38
DXS10074	17	15
DXS10101	30, 31	30.2
DXS10135	23, 26	33

### Autosomal SNPs analysis

In this study, the full 40 SNP panel was run on DNA derived from the putative mother and the child. As illustrated in Table 3, there are five independent loci with opposite homozygous genotypes. Because the average mutation frequency is 1 in 10^6^ for a given SNP locus, there would be approximately a 1 in a million probability for a child to have an allele that is inconsistent with the mother’s genotype at a single locus and less than a 1 in 10^30^ probability of having five loci inconsistent with the biological mother’s genotype. These data clearly exclude the putative mother from being the biological mother of the child.

**Table 3:** Genotyping results of 40 autosomal SNP loci

SNP locus	Mother (M)	Boy (B)	SNP locus	Mother (M)	Boy (B)
rs 10092491	C	C	rs 2567608	A	AG
rs 1019029	CT	T	rs 279844	AT	A
rs 10488710	C	CG	rs 315791	AC	C
rs 1058083	AG	A	rs 321198	C	CT
rs 1109037	A	G	rs 338882	T	CT
rs 12997453	G	G	rs 3780962	CT	T
rs 13134862	AG	AG	rs 445251	C	C
rs 13182883	AG	AG	rs 447818	G	AG
rs 13218440	AG	A	rs 560681	AG	AG
rs 1336071	AG	AG	rs 6444724	CT	CT
rs 1358856	A	A	rs 6591147	CT	CT
rs 1410059	C	CT	rs 6811238	GT	G
rs 1478829	T	AT	rs 7205345	CG	CG
rs 1523537	CT	T	rs 7229946	G	A
rs 1554472	T	T	rs 740598	G	G
rs 1821380	CG	G	rs 7520386	AG	A
rs 2073383	CT	C	rs 7704770	A	A
rs 214955	G	A	rs 985492	C	T
rs 2272998	CG	CG	rs 987640	T	T
rs 2503107	A	AC	rs 9951171	G	A

### Mitochondrial DNA analysis

In this study, mitochondrial profiles were derived from the putative mother and child and compared. As illustrated in Table 4, there are clear and obvious differences between the two profiles, further corroborating the exclusion suggested by the X-STR and SNP data above. It is apparent that the child has considerable C-length heteroplasmy in HV1 and HV2 which is not apparent in the profile of the putative mother. For example, for primer pair 2896 which covers coordinates 16102..16224, a single base composition of A45 G13 C41 T24 is observed while the same primer pair yields multiple length variants of these coordinates spanning four C-length variants with base compositions of A44 G13 *C43* T22 to A44 G13 *C46* T22. Note also that the putative mother has 45 A’s and 24 T’s over these coordinates and the child, regardless of C-length variation, consistently has 44 A’s and 22 T’s. These differing base compositions represent multiple clear and unique differences between the mitochondrial profiles of the child and the putative mother over the coordinates spanned by a single primer pair. Perhaps more importantly, there are clear and distinct base composition differences in 9 of the 24 primer pairs, clearly and unambiguously inconsistent with a profile shared between child and biological mother.

**Table 4:** Typing results of mt DNA HV1 and HV2

mt DNA HVI			mt DNA HVII		
Primer	Segment	Base composition	Primer	Segment	Base composition
2901	15893..16012	M: A47 G18 C25 T30	2902	5..97	M: A19 G24 C24 T26
		B: A47 G18 C25 T30			B: A19 G24 C24 T26
2925	15937..16041	M: A35 G14 C24 T32	2903	20..139	M: A24 G34 C29 T33
		B: A35 G14 C24 T32			B: A24 G34 C29 T33
2899	15985..16073	M: A26 G15 C21 T27	2904	83..187	M: A23 G21 C29 T32
					M: A23 G21 C30 T31
		B: A26 G15 C21 T27			B: A23 G21 C29 T32
2898	16025.0.16119	M: A26 G17 C27 T25	2905	113..245	M: A39 G18 C28 T48
					M: A39 G18 C29 T47
		B: A26 G17 C27 T25			B: A39 G18 C27 T49
					B: A39 G18 C28 T48
2897	16055..16155	M: A31 G13 C29 T28	2906	154..290	M: A48 G18 C31 T40
		B: A31 G13 C29 T28			B: A48 G18 C31 T40
2896	16102..16224	M: A45 G13 C41 T24	2908	204..330	M: A42 G16 C38 T32
		B: A44 G13 C43 T22			B: A42 G16 C39 T32
		B: A44 G13 C44 T22			B: A42 G16 C40 T32
		B: A44 G13 C45 T22			
		B: A44 G13 C46 T22			
2895	16130..16224	M: A36 G7 C33 T19	2907	239..363	M: A44 G10 C49 T23
		B: A35 G7 C34 T18			B: A43 G11 C50 T23
		B: A35 G7 C35 T18			B: A43 G11 C51 T23
		B: A35 G7 C36 T18			
		B: A35 G7 C37 T18			
2893	16154..16268	M: A44 G7 C45 T19	2923	262..390	M: A47 G10 C53 T20
		B: A43 G7 C48 T16			B: A47 G10 C54 T20
		B: A43 G7 C49 T16			B: A47 G10 C55 T20
		B: A43 G7 C50 T16			
		B: A43 G7 C51 T16			
2892	16231..16338	M: A40 G9 C39 T20	2910	331..425	M: A33 G9 C27 T26
		B: A41 G8 C40 T19			B: A33 G9 C27 T26
2891	16256..16366	M: A38 G8 C40 T25	2916	367..463	M: A27 G8 C32 T30
		B: A37 G9 C41 T24			B: A27 G8 C32 T30
2890	16318..16402	M: A20 G14 C31 T20	2912	409..521	M: A32 G7 C48 T26
		B: A20 G14 C30 T21			B: A32 G7 C48 T26
2889	16357..16451	M: A21 G17 C36 T21	2913	464..603	M: A43 G10 C62 T23
		B: A21 G17 C36 T21			B: A44 G10 C63 T23

### Discussions and conclusions

Forty-six autosomal STRs, 20 X-STRs, 40 SNPs, and mtDNA were typed for the resolved case. Calculated on the basis of population genetics data [7–10, 14], in Chinese Han population, the accumulative exclusion power of the 46 autosomal loci and 20 X-STR markers in duos was 0.999999999999986 and 0.999999948, respectively.

The data presented in Table 2, 3 and 4, taken in aggregate, clearly exclude the putative mother from being the child’s biological mother. Subsequent to these studies, it was determined that the putative mother was in fact a full sibling of the boy’s biological father; that is to say the putative mother was in fact the boy’s aunt—not his biological mother.

This case warns that there will be instances when strong DNA evidence will lead to an incorrect conclusion, especially in cases with an unknown family background. von Wurmb-Schwark once reported the possible pitfalls in deficiency cases [15]. According to his report, if the alleged parent and the true parent are full siblings, the false inclusion rate may be as high as 4 % using the AmpFℓSTR^®^ Identifiler^®^ kit, which amplifies 15 autosomal STRs simultaneously. Therefore, it is clearly important to increase the number of investigated loci or include a typing of sex chromosome specific STRs to further ascertain the results. It is particularly worth mentioning that X-STRs would have been a quick way to exclude relationships and very powerful in some deficiency cases (as well as incest cases), even though the power of discrimination of the X-STRs is less than the autosomal STRs [15, 16]. As shown in this work, X-STR markers were immediately able to exclude the false mother.

Besides, autosomal SNPs and mtDNA could also be used to complement autosomal STR typing if there is a possibility of the putative mother being genetically related to the biological parents of the child. The 40-locus binary markers detected in the case were originally selected by Kidd and co-workers [3]. This 40 SNP panel is expected to have an average random match probability of ~1 × 10^−15^. As these markers are robust in terms of stability of inheritance, it serves as a useful tool, orthogonal to STRs. As for mtDNA, due to maternal inheritance, the marker is valuable for testing of relationships between maternal individuals. Although the PLEX-ID platform for analyzing of SNP and mtDNA in this case is now no longer available, the comparative analysis of the PLEX-ID technology and the traditional capillary electrophoretic system for typing of amplified DNA fragments has demonstrated the potential advantages of the mass-spectrometric technique [17].

## Abbreviations

ESI-TOF-MS: electrospray ionization time-of-flight mass spectrometry
HV1: hypervariable region I
HV2: hypervariable region II
mtDNA: mitochondrial DNA
PCR: polymerase chain reaction
SNP: single nucleotide polymorphism
STR: short tandem repeat
X-STR: X-chromosomal short tandem repeat
